# In an *in vitro* model of human tuberculosis, monocyte-microglial networks regulate matrix metalloproteinase-1 and -3 gene expression and secretion via a p38 mitogen activated protein kinase-dependent pathway

**DOI:** 10.1186/1742-2094-10-107

**Published:** 2013-08-26

**Authors:** Justin A Green, Lucinda Rand, Rachel Moores, Shruti Dholakia, Theodore Pezas, Paul T Elkington, Jon S Friedland

**Affiliations:** 1 Section of Infectious Diseases and Immunity and the Imperial College Wellcome Trust Centre for Clinical Tropical Medicine, Hammersmith Campus, Imperial College London, London, W12 0NN, UK; 2 Currently: Clinical and Experimental Sciences, University of Southampton, Southampton, SO16 6YD, UK

**Keywords:** Tuberculosis, Central nervous system, Matrix metalloproteinase, Immunopathology, Mitogen-activated protein kinase

## Abstract

**Background:**

Tuberculosis (TB) of the central nervous system (CNS) is characterized by extensive tissue inflammation, driven by molecules that cleave extracellular matrix such as matrix metalloproteinase (MMP)-1 and MMP-3. However, relatively little is known about the regulation of these MMPs in the CNS.

**Methods:**

Using a cellular model of CNS TB, we stimulated a human microglial cell line (CHME3) with conditioned medium from *Mycobacterium tuberculosis*-infected primary human monocytes (CoMTb). MMP-1 and MMP-3 secretion was detected using ELISAs confirmed with casein zymography or western blotting. Key results of a phospho-array profile that detects a wide range of kinase activity were confirmed with phospho-Western blotting. Chemical inhibition (SB203580) of microglial cells allowed investigation of expression and secretion of MMP-1 and MMP-3. Finally we used promoter reporter assays employing full length and MMP-3 promoter deletion constructs. Student’s *t*-test was used for comparison of continuous variables and multiple intervention experiments were compared by one-way ANOVA with Tukey’s correction for multiple pairwise comparisons.

**Results:**

CoMTb up-regulated microglial MMP-1 and MMP-3 secretion in a dose- and time-dependent manner. The phospho-array profiling showed that the major increase in kinase activity due to CoMTb stimulation was in p38 mitogen activated protein kinase (MAPK), principally the α and γ subunits. p38 phosphorylation was detected at 15 minutes, with a second peak of activity at 120 minutes. High basal extracellular signal-regulated kinase activity was further increased by CoMTb. Secretion and expression of MMP-1 and MMP-3 were both p38 dependent. CoMTb stimulation of full length and MMP-3 promoter deletion constructs demonstrated up-regulation of activity in the wild type but a suppression site between -2183 and -1612 bp.

**Conclusions:**

Monocyte-microglial network-dependent MMP-1 and MMP-3 gene expression and secretion are dependent upon p38 MAPK in tuberculosis. p38 is therefore a potential target for adjuvant therapy in CNS TB.

## Background

Tuberculosis (TB) remains a deadly disease responsible for approximately 1.5 million deaths a year, many caused by disseminated disease involving the central nervous system (CNS) [1]. During active disease, infection of monocytic cells by *Mycobacterium tuberculosis (M.tb)* results in an intense host-driven inflammatory response that contributes not only to pathogen death, but also to local tissue destruction and immunopathology. In CNS TB, this inflammatory reaction is not well-tolerated and is responsible for infarction, hydrocephalus, neuronal damage and death.

Monocytic cell-derived matrix metalloproteinases (MMPs) are central mediators of TB-driven pulmonary tissue destruction due to their ability to degrade fibrillar collagens [2]. Indeed this family of 23 proteases with differing, but overlapping, substrates can degrade all elements of the extracellular matrix at neutral pH, differentiating them from other proteases that might also be induced in inflammatory situations [3,4]. MMPs perform multiple roles in development, reproduction and the normal immune response, such as facilitating leucocyte recruitment, processing cytokines and chemokines, activating defensins and remodelling matrix [5]. In the CNS, MMPs are known to have a number of substrates, such as dystroglycan, a transmembrane receptor that anchors astrocyte endfeet to the basement membrane via laminin binding [6]. Cleavage of these molecules contributes to blood-brain barrier breakdown. The resident CNS innate immune cell is the microglia, most likely of mesodermal monocyte-lineage origin [7]. We and others have previously shown that microglia secrete high concentrations of MMPs in CNS TB [8-10]. MMP-1, MMP-3, MMP-8 and MMP-9 are found at high concentrations in the cerebral spinal fluid (CSF) of CNS TB patients [11]. In addition, MMP-1, MMP-3 and -9, but not the specific tissue inhibitor of metalloproteinase (TIMP) -1, are present in CNS tuberculomas [9,12]. Specifically MMP-1 is an interstitial collagenase able to digest aggrecan and collagen at neutral pH, and MMP-3 is a stromelysin with substrates such as fibronectin, laminin and type IV collagen [3]. Finally dexamethasone, a specific and proven adjuvant to anti-tuberculous chemotherapy that improves patient outcomes, specifically suppresses MMPs, but not TIMPs, in CNS TB patients [11,13].

Intracellular signalling is critical to control of MMP transcription and secretion. We have shown that nuclear factor-κB (NF-κB) and activator protein (AP)-1 pathways are activated both *in vitro* and in patients with CNS TB [9]. Furthermore, the bacteriostatic TB drug para-aminosalicyclic acid exerts its anti-TB activity in part due to inhibition of the prostaglandin pathway, thereby reducing MMP-1 secretion and tissue destruction [14]. Upstream of these signalling pathways are the mitogen-activated protein kinases (MAPKs). The three main pathways are p38, c-Jun N-terminal kinase (JNK) and extracellular signal-regulated kinase (ERK)-1/2. The extracellular signal-regulated MAPK are involved in cell turnover and phosphorylation of transcription factors and microtubule-associated proteins, whereas the p38 MAP kinase subfamily regulates cell differentiation, apoptosis and cellular response to inflammation. p38 MAPK may be an important point of divergence in the regulation of MMP and TIMP secretion in pulmonary TB [15]. In the CNS, MMP-9 secretion by astrocytes was dependent upon an IL-1β/MAP kinase/AP-1 pathway [16].

In this study, we tested the hypothesis that MAPK pathways are key factors in the control of microglial MMP gene expression and secretion using a cellular model of CNS TB. We present evidence that the p38 pathway is the principle regulator of MMP secretion in microglia, causing divergent secretion of MMP-1 and MMP-3 and their associated TIMPs.

## Methods

### Reagents

Chemicals were from Sigma-Aldrich (Gillingham, UK), tissue culture materials from Invitrogen (Paisley, UK) and tissue culture plastic from TPP (Trasadingen, Switzerland) unless otherwise stated. Inhibition experiments were performed with a two-hour pre-incubation of the specific p38 chemical inhibitor SB203580 from Promega (Southampton, UK) at concentrations as shown.

### *M.tb* culture

The virulent *M.tb* strain H73Rv Pasteur was cultured in Middlebrook 7H9 broth (BD Biosciences, Oxford, UK) with 10% OADC enrichment medium, 0.2% glycerol and 0.02% Tween 80 with agitation. *M.tb* at optical density (OD) 0.6 (Biowave Cell Density Meter; WPA, Cambridge, UK), corresponding to 10^8^ colony forming units/ml (cfu/ml) was used for infection. Endotoxin level was <0.03 ng/ml lipopolysaccharide (LPS) (Amoebocyte lysate assay, Associates of Cape Cod, Falmouth, MA, USA).

### Cell culture and *M.tb* infection

Monocytes were isolated from healthy blood donors (UK Blood Transfusion Service) by Ficoll-Paque (GE Healthcare, Little Chalfont, UK) density gradient centrifugation and adherence purification [17]. Monocytes were infected with *M.tb* at a multiplicity of infection of 1. At 24 hours, cell culture medium was collected and 0.2 μm was sterile-filtered (Nalgene, Hereford, UK) to remove infectious particles but not MMPs [18]. Conditioned medium from *M.tb*-infected monocytes was termed CoMTb and control medium from uninfected monocytes was CoMCont.

Human CHME3 microglial cells (used with permission of Marc Tardieu, Paris, France and kindly provided by Nicola Woodroofe, Sheffield Hallam University, UK) were maintained in DMEM with 10% FCS (Biosera, Ringmer, UK) and 3 mM glutamine. Cells were seeded at 35,000 to 50,000 cells/cm^2^ for experiments, which were performed in macrophage serum-free medium (MSFM).

### RNA extraction, cDNA synthesis and quantitative reverse-transcribed PCR (qRT-PCR)

Microglia were lysed with TRI reagent and total RNA was extracted. cDNA was prepared from 1 ug RNA and reverser transcribed using 2 μl of random primers and 200 units SuperScript III reverse transcriptase (both Invitrogen), according to supplier’s instructions. Quantitative PCR (qPCR) was performed as previously described [9]. The cycle threshold (Ct) at which amplification entered the exponential phase was determined and this number was used to indicate the amount of target RNA in each sample. MMP Cts were normalized to ribosomal 18S mRNA.

### Casein zymography

We loaded 20 μl of cell culture medium with 5 μl of loading buffer (0.25 M Tris pH 6.8, 50% glycerol, 5% SDS and bromophenol blue crystals) on 12% casein gels (Invitrogen). Gels were run at 125 V for 2.5 hours (buffer 25 mM Tris, 190 mM glycine and 0.1% SDS). After one hour incubation in 2.5% Triton-X, gels were transferred to collagenase buffer (55 mM Tris base, 200 mM NaCl, 5 mM CaCl_2_ and 0.02% Brij; pH 7.6) for 40 hours at 37°C. Gels were stained with 0.2% Coomassie blue (Pharmacia Biotech, Sweden) for one hour before de-staining in acetic acid/methanol/water (1:3:6), and 5 ng of MMP-1 standard (Merck, Germany) was run on each gel. Gels were analyzed densitometrically by Scion Image Analysis (NIH Image version 1.61, Bethesda, MD, USA) normalized to standard.

### Western blotting

Standard MMP-1 western blotting was performed as described [19]. Overnight incubation in 1:1,000 MMP-1 primary antibody (The Binding Site, Birmingham, UK) in 5% milk protein was followed by washing and incubation with 1:2000 peroxidase-conjugate secondary antibody for an hour (The Binding Site). Bands were visualized by chemiluminescence with the ECL Plus system (GE Healthcare) according to manufacturer’s instructions. MMP-3 western blotting was performed using 1:5,000 rabbit anti-MMP-3 primary antibody (Chemicon, Millipore, Watford, UK) in 5% bovine serum albumin and 1:2,000 horseradish peroxidase (HRP)-linked anti-rabbit secondary (Cell Signalling Technology, Danvers, MA, US) in 5% milk protein. For MAPK experiments, cells were lysed using 200 μl of SDS sample buffer (62.5 mM Tris pH 6.8, 2% SDS, 10% glycerol, 50 mM DTT and 0.01% bromophenol blue) and immediately frozen at −80°C. Then, 40 μl aliquots were run on western gels as described and probed with 1:1,000 rabbit primary antibodies to both phosphorylated and unphosphorylated forms of p38 or ERK MAPK. 1:2,000 dilution of anti-rabbit HRP-linked secondary antibody was used (all Cell Signalling Technology).

### Luminex analysis

MMP-1 and MMP-3 concentrations were analyzed by Fluorokine MAP profiling kit according to the manufacturer’s protocol (R&D systems) on the Luminex platform (Biorad, Hemel Hempstead, UK). The minimum level of detection for MMP-1 was 10 pg/ml and 15 pg/ml for MMP-3.

### MMP-3, TIMP-1 and -2 ELISAs

MMP-3 and TIMP-1 and TIMP-2 concentrations in cell culture medium were measured by ELISA (R&D Systems) according to the manufacturer’s instructions. The lower limit of detection was 30 pg/ml.

### Phospho-MAPK array

The Proteome Profiler phospho-array (R&D systems) was used to investigate multiple kinase pathways. We stimulated 10^6^ microglia with CoMCont or CoMTb for 30 minutes. The phospho-array was performed according to the manufacturer’s protocol and developed with the ECL system (GE Healthcare). Densitometric analysis of each array was performed using NIH Image version 1.61. The Proteome Profiler kit measured relative MAPK and related serine/threonine kinase phosphorylation status. Six positive controls are incorporated onto the membrane. Mean intensity was calculated for CoMCont and CoMTb. Data are presented as the difference between these mean intensities.

### Promoter reporter analysis

Microglial cells were co-transfected with MMP-3 promoter constructs and a control reporter plasmid, constitutively expressing Renilla luciferase, with Fugene HD (Roche). Cells were stimulated after 18 hours, and luciferase activity analyzed 24 hours later by the Dual-Luciferase Reporter Assay System (Promega) on a luminometer (Medical Devices, Wokingham, UK). Renilla luciferase activity was used to normalize firefly luciferase activity to control for transfection efficiency.

### Statistics

Data are presented as means (± SD) of three samples and represent experiments performed in triplicate on at least two occasions, unless otherwise stated. Paired groups were compared with Student’s *t*-test. Multiple intervention experiments were compared by one-way analysis of variance (ANOVA) and Tukey’s correction for multiple pairwise comparisons with equal variance tested by Levene’s test. *P* <0.05 was taken as significant. All analysis was done using SPSS software (version 15.0, SPSS corp., Chicago, IL, USA).

## Results

### Monocyte-microglial networks drive MMP-1 and MMP-3 secretion from human microglia

**Figure 1:**
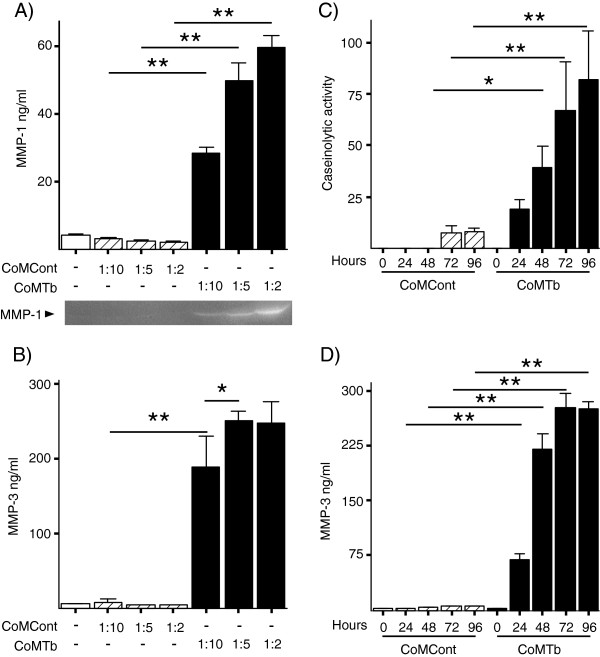
**Matix metalloproteinsase (MMP)-1 and MMP-3 secretion is up-regulated by conditioned media from tuberculosis (TB)-infected monocytes (CoMTb) in a dose- and time-dependent fashion. (A and B)** Microglial cells were stimulated with control medium (open bars), or increasing concentrations of conditioned media from control monocytes (CoMCont) (diagonal hatched bars) and CoMTb (solid bars); 72-hour supernatants were analyzed by Luminex for MMP-1 or MMP-3. A representative casein zymogram is also shown **(A)**. Kinetics of MMP-1 activity **(C)** and MMP-3 secretion **(D)** demonstrate progressive increase in concentration for up to 72 hours. Bars represent mean values ± SD of three samples, representative of at least duplicate experiments performed in triplicate. ^*^P <0.05, ^**^P <0.01.

Increasing concentrations of CoMTb but not CoMCont resulted in greater secretion of MMP-1 and MMP-3 in a dose-dependent manner (Figure 1A and B). Specifically, MMP-1 secretion showed a stepwise increase in secretion 9.2-fold higher at the 1:10 dilution of CoMTb compared to CoMCont and reaching 27.9-fold greater at 1:2 dilutions (all *P* <0.01). MMP-3 secretion was 12-fold higher at the 1:10 dilution of CoMTb than the equivalent CoMCont concentration and 58-fold at the 1:2 dilution (*P* <0.01). Since there was no further increase in MMP-3 secretion after a 1:5 dilution of CoMTb, this concentration was subsequently used to stimulate cells in all experiments. MMP-1 and MMP-3 kinetic experiments demonstrated a significant difference in MMP secretion in response to CoMTb compared to CoMCont (Figure 1C and D). MMP-1 activity was detectable at 24 hours and maximal at 96 hours with a 5.3-fold change from CoMCont (*P* <0.01). MMP-3 secretion peaked at 72 hours with a 58-fold increase, but there was no further increase at 96 hours (*P* <0.01). Therefore, cell culture supernatants were harvested at 72 hours in subsequent experiments.

### Phospho-array analysis of CoMTb-stimulated microglia

**Figure 2:**
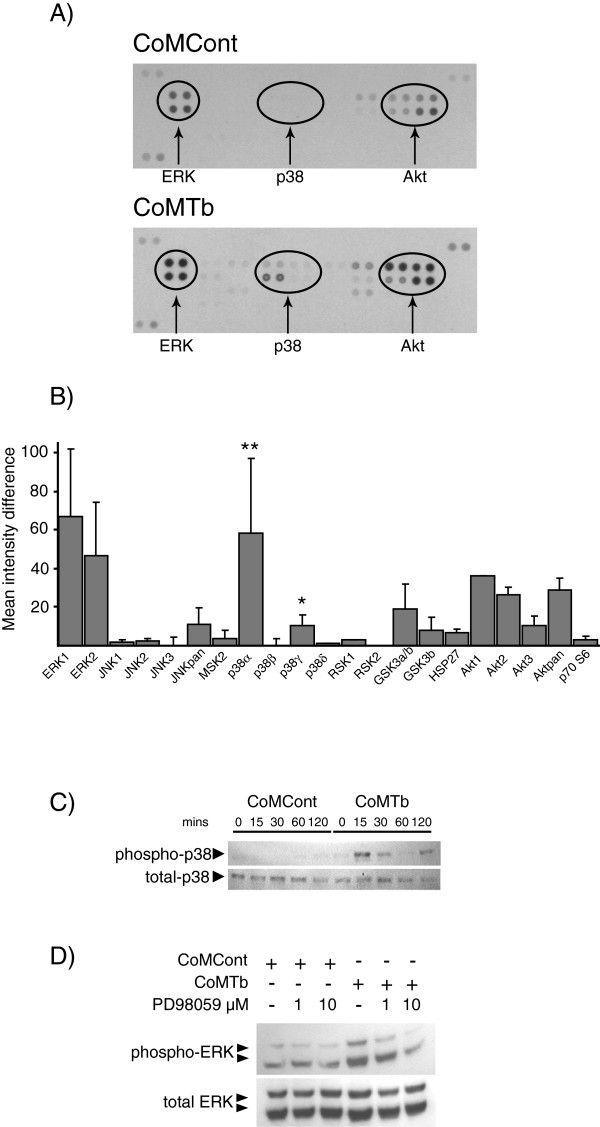
**Conditioned media from tuberculosis (TB)-infected monocytes (CoMTb) drive phosphorylation of the extracellular signal-regulated kinase (ERK), p38 and AKT mitogen-activated protein kinase (MAPK) pathways in microglial cells. (A)** Proteome profiler array of conditioned media from control monocytes (CoMCont)- and CoMTb-stimulated microglia at 30 minutes. CoMTb drives p38 activity and further phosphorylation of constitutive ERK 1/2 and AKT activity. **(B)** Densitometric analysis of phospho-array. Densitometric analysis of differences in mean spot intensity between CoMCont- and CoMTb-stimulated cells is presented, demonstrating that the p38α and p38γ subunits are the principal CoMTb up-regulated MAP kinases. **(C)** p38 phosphorylation kinetics. CoMTb drives up-regulation of p38 at 15 minutes, with a second peak at 120 minutes. **(D)** CoMCont-stimulated microglial ERK activity at 30 minutes shows high basal activity further up-regulated by CoMTb. Only CoMTb activity was blocked by the inhibitor PD98059. Representative data from three independent experiments are shown. Bars represent mean +/− standard error of the mean from three independent experiments. ^*^P <0.05, ^**^P <0.01.

Next, we investigated the intracellular signalling pathways regulating MMP-1/3 secretion using phosphor-array screening. The ERK and AKT pathways were activated in CoMCont-stimulated cells. At 30 minutes, CoMTb-stimulated multiple signalling pathways including ERK, p38, AKT, GSK, JNK and HSP-27 (Figure 2A). Densitometric analysis of differences in mean spot intensity between stimulated and unstimulated cells demonstrated that ERK-1/2 and AKT phosphorylation were up-regulated to the greatest extent in CoMTb-stimulated cells (Figure 2B). The increases in different p38 isoforms were divergent. CoMTb stimulated p38α phosphorylation 311% more than CoMCont (*P* <0.05) and p38γ phosphorylation increased by 154% (*P* <0.05). p38β and p38δ phosphorylation did not change.

To investigate phospho-array findings further, kinetic Western blot analysis of CoM-stimulated p38 phosphorylation in microglia was performed over 120 minutes (Figure 2C). Minimal basal phosphorylation was seen. CoMTb stimulated p38 phosphorylation at 15 minutes, which waned by 60 minutes. However, a second peak of phosphorylation was detected at 2 hours. At 30 minutes, basal ERK phosphorylation was detected. Although CoMTb up-regulated ERK phosphorylation was blocked by PD98059 in a dose-dependent manner, the basal phosphorylation made subsequent interpretation of the effect of ERK hard to interpret, and so it was not studied further (Figure 2D).

### Regulation of MMP-1 and MMP-3 secretion by p38

**Figure 3:**
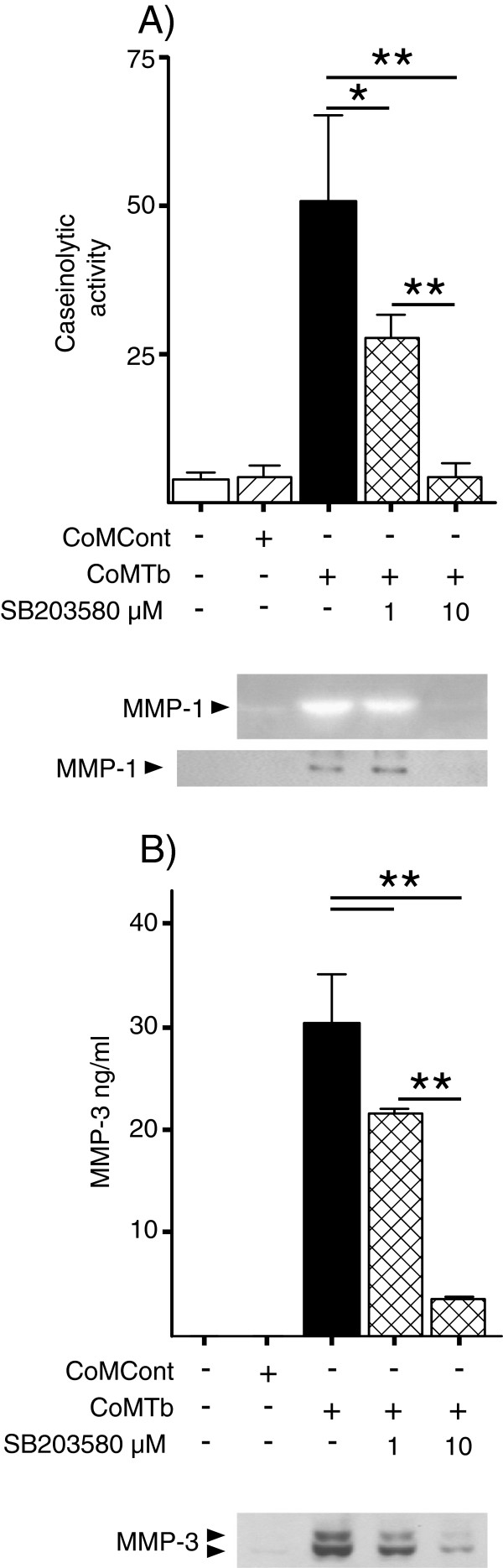
**p38 phosphorylation controls both matix metalloproteinsase (MMP)-1 and MMP-3 secretion in conditioned media from tuberculosis (TB)-infected monocytes (CoMTb)-stimulated microglia.** Microglial cells were incubated with SB203580 for 2 hours, and then stimulated with CoMTb. **(A)** MMP-1 activation and secretion was analyzed at 72 hours by casein zymography (representative gel shown) and western blotting. **(B)** MMP-3 activity was analyzed by ELISA and western blotting. p38 inhibition suppressed CoMTb MMP-1 and MMP-3 suppression in a dose-dependent manner. Bars represent mean values ± SD of three samples, representative of at least duplicate experiments performed in triplicate. Data were analyzed by one-way analysis of variance, followed by Tukey’s multiple comparison test. ^*^P <0.05, ^**^P <0.01.

To dissect the role of the p38 pathway in control of MMP-1 and MMP-3 secretion, a specific chemical inhibitor (SB203580) was pre-incubated with microglia prior to CoMTb stimulation. MMP-1 secretion was suppressed by 45.6% by 1 μM and 92% by 10 μM SB 203580 (*P* <0.05 and *P* <0.01 respectively) (Figure 3A). MMP-3 was also p38-dependent; 1 and 10 μM SB203580 reduced MMP-3 secretion by 28.8% and 83.2% respectively (both *P*<0.01) (Figure 3B).

**Figure 4:**
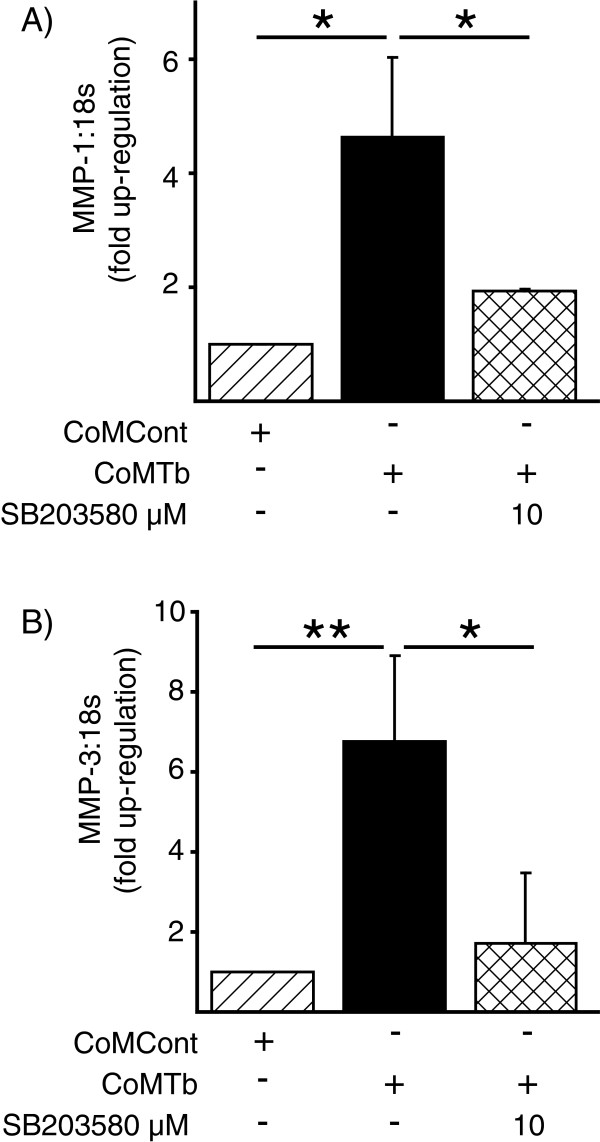
**Matrix metalloproteinase (MMP)-1 and MMP-3 gene expression is suppressed by p38 inhibition. (A)** Shows MMP-1 and **(B)** shows MMP-3 gene expression. mRNA from microglia stimulated for 24 hours with conditioned media from tuberculosis (TB)-infected monocytes (CoMTb) was analyzed by real-time PCR, normalized to 18S RNA and expressed as fold-change relative to mRNA from 24 hour-conditioned media from control monocytes (CoMCont). The mean ± SD from three experiments are shown. Data were analyzed by one-way analysis of variance, followed by Tukey’s multiple comparison test. ^*^P <0.05, ^**^P <0.01.

MMP-1 and MMP-3 gene expression were investigated by qRT-PCR. MMP-1 gene expression was up-regulated by CoMTb (Figure 4A). This up-regulation was suppressed by inhibition of the p38 MAPK. MMP-3 mRNA accumulation was also decreased by p38 inhibition (Figure 4B), consistent with secretion data.

### The MMP-3 promoter is up-regulated by CoMTb and has a suppression site between −2183 and −1612 bp

**Figure 5:**
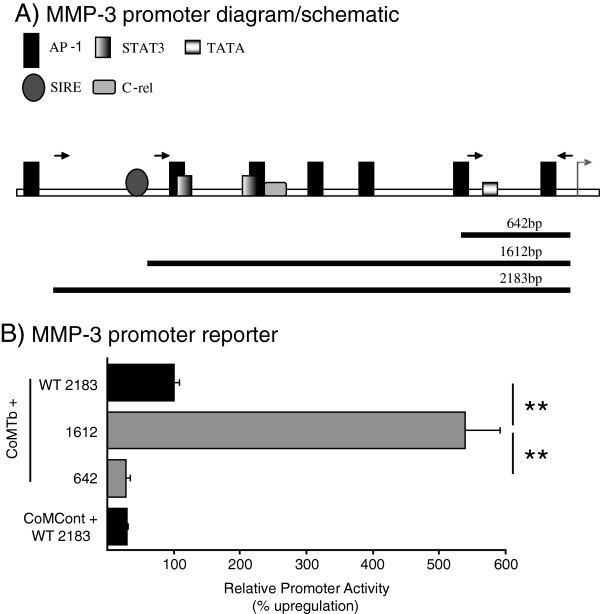
**Matrix metalloproteinase (MMP)-3 promoter activity. (A)** Schematic representation of full-length MMP-3 promoter demonstrating relevant promoter binding sites (shaded boxes). Right to left arrow represents transcription start site. Left to right arrows represent transcription stopping sites on the three promoter constructs, for which the length is indicated by the black line below. Of note, the 1612 bp construct disrupts a stromelysin IL-1 responsive element (SIRE) binding site. **(B)** MMP-3 promoter deletion constructs. Microglial cells were transiently transfected with full-length promoter (black bars) or deletion constructs (gray bars), and stimulated with, conditioned media from control monocytes (CoMCont) or conditioned media from tuberculosis (TB)-infected monocytes (CoMTb) for 24 hours. CoMTb-stimulated full-length MMP-3 promoter was designated as maximal luciferase activity (100%), and other results are expressed as a percentage of this maximum. Bars are mean ± SD from three samples and represent at least three separate experiments performed in triplicate. Data were analyzed by the Student *t*-test (^**^P <0.01).

The MMP-3 promoter has multiple transcription factor binding sites (Figure 5A) and has not been previously investigated in CNS TB. MMP-3 promoter reporter analysis demonstrated that CoMTb increased the 2183 bp full-length promoter activity above CoMCont levels by 3.4-fold at 24 hours (Figure 5B, *P* <0.05). A significant rise in promoter activity was seen with the 1612 bp promoter compared to the full length promoter in response to CoMTb stimulation (*P* <0.01). Promoter activity was decreased compared to CoMCont levels when the shorter 642 bp construct was stimulated with CoMTb, suggesting that this construct does not contain the minimal elements necessary to allow promoter activity in response to CoMTb.

## Discussion

In this study, we have demonstrated that MMP-1 and MMP-3 secretion from monocyte-microglial networks are dependent on the p38, MAPK pathway in a cellular model of CNS TB. This control acts, at least in part, at the transcriptional level. We also defined that a key repressor element exists in the MMP-3 promoter upstream of −1612 from the transcriptional start site.

First, we delineated that CoMTb, but not CoMCont, drives dose-dependent MMP-1 and MMP-3 secretion. This effect peaked for MMP-3 at 72 hours, but MMP-1 secretion increased until 96 hours. Next, using phosphor-arrays, we demonstrated that the ERK and AKT pathways were active in CoMCont-stimulated cells. These pathways are responsible for cell turnover, and therefore, basal expression in cell lines is commonly found [20]. Second, although there was increased phosphorylation of these pathways with CoMTb, the p38 pathway, and in particular the p38α and γ subunits, stood out as principle targets of CoMTb-activated signaling. This was confirmed in kinetic studies where biphasic phosphorylation of p38 was observed. Biphasic p38 activation is linked to the apoptotic pathway, which is activated only after the second peak in phosphorylation [21]. No specific subunit analysis was performed in our experiments, because the antibody used recognizes epitopes common to all four p38 subunits. However, *M.tb* has also been shown to directly drive p38 phosphorylation in microglia. Yang *et al*. demonstrated that sonicated (therefore dead) *M.tb* stimulated p38 phosphorylation in a toll-like receptor (TLR)2-dependent manner [22]. In our study, p38α activation was most marked. Conditional knockout of p38α in murine macrophages differentially affects bacterial phagocytosis and LPS-induced TNF-α, but not IL-6, secretion via the attenuation of the transcription factor cAMP response element-binding protein (CREB) but not the NF-κB pathway [23]. There is emerging evidence that p38 inhibition may have a therapeutic role in the treatment of cerebral ischaemia, where it reduces neuronal death and microglial activation suggesting a possible translational application for our data [24]. In addition, we found that MMP-3 was solely p38-dependent, and thus, p38 appears to be a central cellular control point for MMP-1 and MMP-3 secretion in microglial cells.

The high ERK basal phosphorylation up-regulated by CoMTb observed in the MAP kinase array was confirmed by western blotting. However, since in cell lines, high basal ERK activity is driven by the immortalization process, we chose not to study this pathway further. However, ERK activation is known to contribute to the control of MMP secretion in the CNS, macrophages and other cells, suggesting a ubiquitous, rather than specific, cellular role for this MAPK [16,25,26]. Specifically, further experiments in primary cells are warranted to confirm these preliminary findings. Gene expression data exactly reflected secretion data although the extent of any post-transcriptional effect on MMP-1 regulated by p38 was not investigated in the present study.

Finally promoter-reporter studies demonstrated CoMTb-specific up-regulation of promoter activity, and also indicated that a repressor element exists in the upstream part of the promoter (−1612 to −2183). The exact repressor element demonstrated to be acting here is as yet undefined. Tantalizingly the 1612 bp construct exactly disrupts a previously reported SIRE binding site, a transcription factor associated with down-regulation of MMP-3 secretion in fibroblasts [27]. In microglial cells, we have previously proven that the observed CoMTb effect involves synergy between TNF-α and IL-1β for MMP-3 but not MMP-1 secretion, supporting the role of this latter cytokine [9]. However, unlike in respiratory epithelial cells and macrophages, we have been unable to demonstrate an additional effect of *M. tb*-derived antigens on secretion of MMPs or p38 MAPK signalling via TLR2, respectively, in this model system [14,15].

## Conclusions

We have demonstrated that in CNS TB, MMP-1 and MMP-3 secretion from monocyte-microglial networks are critically dependent upon the p38 MAP kinase pathway, and this pathway may warrant further investigation as a therapeutic target in CNS TB.

## Abbreviations

ANOVA: Analysis of variance; AP: Activator protein; bp: Base pairs; cfu: Colony forming units; CoMCont: Conditioned media from control monocytes; CoMTb: Conditioned media from tuberculosis-infected monocytes; CNS: Central nervous system; CREB: cAMP response element-binding protein; CSF: Cerebrospinal fluid; Ct: Cycle threshold; DMEM: Dulbecco’s modified Eagle’s medium; ELISA: Enzyme-linked immunosorbent assay; ERK: Extracellular signal-regulated mitogen-activated protein kinase; FCS: Fetal calf serum; HRP: Horseradish peroxidase; IL: Interleukin; MAPK: Mitogen-activated protein kinase; MMP: Matrix metalloproteinase; MSFM: Macrophage serum-free medium; NF-Κb: Nuclear factor-κB; OD: Optical density; qPCR: Quantitative polymerase chain reaction; SIRE: Stromelysin IL-1 responsive element; TB: Tuberculosis; TIMP: Tissue inhibitor of metalloproteinase; TLR: Toll-like receptor; TNF: Tumour necrosis factor.

## Competing interests

The authors declare no conflict of interest.

## Authors’ contributions

JAG, LR, RM, PTE and JSF designed the experiments, which were undertaken by JAG, RM and SD. TP and LR designed, produced and purified the MMP-3 promoter. JAG wrote the first draft of the manuscript. JAG, LR, RM, PTG and JSF contributed to editing the manuscript. All authors read and approved the final manuscript.
